# Design and Implementation of Human-Computer Interface for Participatory Art Video Development Platform Based on Interactive Non-linear Algorithm

**DOI:** 10.3389/fpsyg.2021.725761

**Published:** 2021-10-28

**Authors:** Xixia Liu, Musen Liu

**Affiliations:** School of Art and Design, Qilu University of Technology (Shandong Academy of Sciences), Jinan, China

**Keywords:** artificial intelligence, online platform, video interaction, artistic composition, art video

## Abstract

Artificial intelligence (AI) technology is innovatively combined with participatory video for artistic creation and communication to improve the enthusiasm of art lovers for artistic creation and communication and expand the application range of AI technology. First, the interactive framework of interactive participation video is proposed based on the analysis of the related literature of interactive non-linear video. Then, a questionnaire is designed accordingly to analyze the social needs of people on art social platforms. According to the survey results, the participatory art video online communication platform is designed and preliminarily realized. Finally, a participant video eye movement control experiment is conducted to test the performance of the participatory art video development platform. Meanwhile, the platform is evaluated through field research from two aspects of test efficiency and user experience. The results show that the operation time of the participatory art video development platform is much shorter than that of the control group. It takes only approximately 15 s to complete the annotation operation with low SD, indicating that the system performance is stable. The accuracy of the platform also reaches 100%.

## Introduction

Nowadays, with the rapid development of science and technology, the integration of art and technology has become increasingly common. The continuous breakthroughs in the application of artificial intelligence (AI) technology make the lives of people more and more intelligent. AI has occupied a place in areas, such as image recognition, speech recognition, intelligent transportation, financial management, medicine, and social networks due to its powerful functionality and adaptability. Lv et al. (2018) proposed a new government service platform using the three-dimensional geographic information system and cloud computing. They conducted three-dimensional analysis and visualization of urban information on the platform of smart cities to effectively manage and use urban data. However, the application of AI in artistic composition and communication is still in an initial stage without deep research (Su and Chiu, 2021; Thomas et al., 2021).

In traditional concepts and artistic composition, the final works of artists are often static. Artworks, such as *Mona Lisa* are suitable for static displays for more mystery. But for other forms of artistic composition, the dynamic display is the more appropriate way to communicate with people (Qian et al., 2018). Dynamic communication can promote ideological collision, inspire inspiration, and promote the development of art and culture. Meanwhile, the composition and communication of artists also need to keep pace with the times through constant communication with the outside world and new knowledge (Dewantara et al., 2021). The development of Internet technology and information technology has created a wide enough communication platform for artists to learn new knowledge or explain their works more conveniently so that audiences can better understand the intention and connotation of their works. In the digital age, the development of applications, such as new media technology has narrowed the distance between artists and audiences, allowing audiences to embrace and feel art, and making art no longer remote.

Participatory video is an application that allows viewers to interact with the video. This research direction of participatory video mainly involves fields of education and social contact. Through the establishment of various social platforms, the audience can interact with the platform through bullet-screen comments (Lin et al., 2019). Better learning and social effects can be achieved by establishing special social relationships on interactive art platforms. AI, as the premise of the mechanical platform with social ability, can assist and optimize the platform functions in many aspects to meet the emotional needs, psychological needs, and functional needs of people in social activities (Rogoza et al., 2018; Harriel and Parboosingh, 2020; Jayanthiladevi et al., 2020).

The innovation and advantage of the research lie in the human-computer interaction (HCI) design of art video development platform integrating AI algorithm with participatory video, which meets the essential social demand and achieves the goal of promoting artistic creation and communication. Based on this, according to the related theory of psychology, a questionnaire is designed to analyze the communication needs of people on the art social platform. Then, a participatory art video interactive platform is designed and preliminarily realized.

## Materials and Methods

### Research on the Participatory Video

Participatory video is also called interactive non-linear video. Interactive non-linear video is a new type of video that uses technical means to integrate the interaction into traditional linear video, which enhances the interactive learning experience. Other researchers believe that participatory video is about the interaction between students and educational content or learning resources in an online learning environment with the achievement of corresponding operations. Feedback plays a mediating role in interactive learning videos. Qian et al. (2018) believed that there is a positive relationship between feedback with task performance, responsibility, and the voice of employees. It is the same for learning. Thus, the interactive behavior in interactive non-linear videos is not only the traditional pause, fast forward, and backward playback but also related to the understanding of the audience and the information transmitted by the video. Interactive video has developed rapidly in education and social networks, but there is minimal research on participatory video in the field of interactive art (Wu et al., 2019; Medusalem et al., 2021). The essence of the interaction is to create value and exchange value. With reference to the design points of interactive videos in educational and social interactions, the participatory video is applied in the interactive learning practice of art.

**FIGURE 1:**
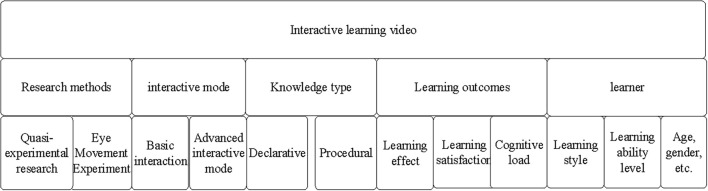
Framework of interactive learning video.

First, teaching videos have become one of the mainstream ways of learning. Therefore, the learning effects of people are closely related to the quality of the selected teaching video (Taslibeyaz, 2020). Novel online educational videos, such as open video lessons, miniature classrooms, and massive open online courses (MOOC), have become a key component of mainstream learning and have brought great convenience to the learning of people. Efficient and fast network information technology has promoted the rise of online courses with an expanding audience range. Through the analysis and summary of relevant literature on interactive non-linear video, the interactive learning video framework is designed as shown in Figure 1.

In Figure 1, the interaction mode in the second part is the focus of the framework of interactive learning video, including basic interaction mode and advanced interaction mode. In the interactive learning video, the basic interaction mode includes forward, pause, and backward, while the advanced interaction mode involves more functions, such as navigation, embedding problem, problem feedback, embedding problem reflection, and hotspot interaction. Most of these interactions occur between people and video interfaces, or between learners and learning contents. The interaction between people occurs less in interactive learning videos, except in live class.

In addition to interactive learning videos, the interactive non-linear video application is also socially oriented. Interactive videos on social platforms and interactive videos in learning have both similarities and differences. The common feature of interactive social video and interactive learning video is the interaction of people with these videos, or the communication of people with other users has created new value (Zhou and Wu, 2018; Cardinale et al., 2020; Patel and Wong, 2020). The difference between them is that interactive videos in interest-based social areas focus on users, based on the sharing of entertainment information between users. The focus of interactive learning videos is the own learning effects of learners and the quality of learning videos.

The depth and breadth of interactive communication is the key research object for the participatory art video communication reported in this study. According to the interactive display mode of information, the socially meaningful participatory art video is innovatively classified into two forms, namely, Internet Relay Chat (IRC) format channel and bullet-screen comments. Bullet-screen comments are real-time comments from viewers flying across the screen like bullets appearing in the video which are visible to every audience. The advantage of the interactive form of bullet-screen comments is that it can make users interact directly and increase the immersion of the audience. IRC is instant messaging software widely used in a group chat or one-to-one chat. In this interactive mode, since the IRC channel is the only form of user interaction, it can highlight the instantaneity of the channel interaction with live video. The interactive information is displayed on the right side of the video so that the main content of the video is not affected by communication. However, interactive information shows that in the chat area, users must enter the dialog box of the chat area when they want to understand interactive information and interactive behavior, which partially reduces the immersion of watching videos.

Based on the above analysis, it is believed that for live games and videos with real-time comments, the importance of the interactive experience of the user is greater than that of the video itself. Therefore, immersion in watching videos is more important. For such a video, the audience does not need to fully participate in video content, and they can choose the interface design with bullet-screen comments. The interactive platform with participatory videos applied to teaching videos and artistic exchanges is more dependent on the quality of videos. Therefore, the content gained by watching the video is more important than the communication between users. Therefore, it is better to choose the IRC interactive mode.

### Influencing Factors of Social Network Based on the Questionnaire Survey

The experience of offline interactive art exhibitions is summarized and used for reference to motivate participants to actively interact with artistic works. It is found that excellent artworks and perfect activity organization mechanisms can greatly stimulate the participation motivation and enthusiasm of participants (Chen, 2019; Sigala and Robinson, 2019). Although the performance media (i.e., photos, texts, and videos) of the Internet platform are different, the forms of performance are almost the same (Jennifer et al., 2020; Yuan et al., 2020). Participants can interact with the works by means of reviewers, bullet-screen comments, and favoriting, while with some artistic interactions (Hearn and Hearn, 2020; Vinken and Upadhye, 2020; Clarabelle et al., 2021). There are also art activities taking online and offline interactions simultaneously with specific processes, using the Internet for publicity while holding offline activities. To a certain extent, this form can encourage art lovers to participate, but the degree and frequency of participation of users are relatively lower than that on entertainment platforms or social platforms. Therefore, many related studies on the network and social psychology are taken as a reference to increase the participation of users of participatory art videos. Wu and Song (2019) studied the satisfaction of social media use in entrepreneurial courses from the perspective of learners and found four satisfactory factors in online entrepreneurial groups, including trust, profit, learning, and socializing. Referring to the classical hierarchical theory of needs proposed by American psychologists Abraham H. Maslow and corresponding literature, a multidimensional questionnaire is designed to analyze the factors affecting the social behavior of participants (Ye et al., 2020). Active users over 3 months from three popular APPs are selected as research objects. A total of 550 questionnaires are distributed through Internet platforms, E-mail, or other channels, and 509 valid questionnaires are returned, with a recovery rate of 92.5%. The efficient rate is 97.6% after excluding 12 questionnaires that are filled incompletely or disorderly. There are 497 effective samples after screening the invalid questionnaires with a relatively large sample size and certain reliability. Respondents cover all age levels, including young and middle-aged people and a small number of elderly people. The gender ratio of respondents is close to 1:1, and nearly 80% of respondents have a bachelor’s degree or above, with closely 30% in art-related majors. Due to the randomness of the questionnaire survey, the low proportion of art major participants is the defect that needs to be improved in this survey, which may also become a factor affecting the results.

### Design of Online Participatory Art Video Platform

**FIGURE 2:**
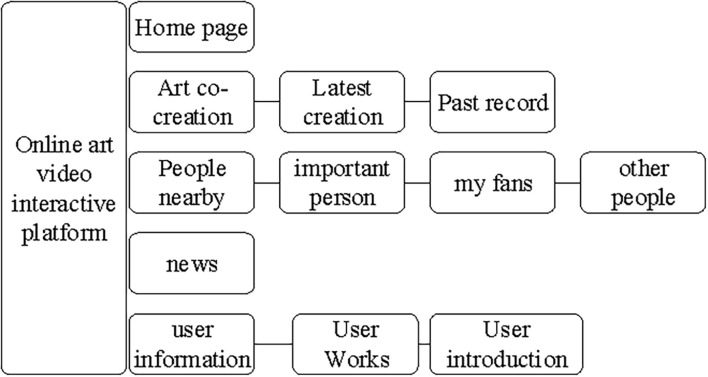
Architecture of the online platform.

According to the analysis results of the questionnaire, the architecture of the online participatory art video platform is designed as shown in Figure 2.

In Figure 2, the platform consists of five parts, namely, the homepage, art co-composition, nearby users, new messages, and user information. Users can enter the user information module to set their own profile photo or information, upload artworks and descriptions of works, and save inspiration (Makoto et al., 2020). The new message part mainly accepts information from the entire online platform. The module of nearby users displays neighboring users on the online participatory art video platform, which is conducive to friendly offline communication among users, allowing users to selectively expand their social circle. The art co-composition part is the site provided by the platform for users to communicate. Users can upload their latest works to the website for communication and learning with others. They can also click on the previous content to view the communication between themselves and other authors in the past and obtain creative experience and inspiration.

**FIGURE 3:**
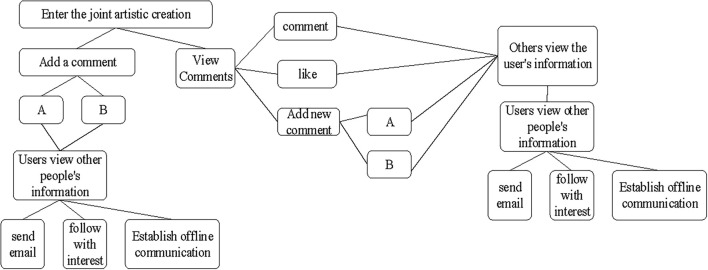
Main operations and interactive relationship chains (A represents comments visible only to the author and the publisher; B represents comments open to all users).

In this study, the interactive art website designed is primarily aimed at activity participants and artists. The limitation of website design and development is manifested in the project cycle, and there may be some loophole update problems in future. According to the architecture shown in Figure 2, the key part of modular website design based on interactive consciousness chain theory includes the following three parts: the development of personal files, the design of art co-composition part, and the design of nearby users. The main operations and interactive relationship chains of these three parts are shown in Figure 3.

According to the interactive relationship chain shown in Figure 3, the online participatory art video platform also includes the offline social part. Every user on the platform has the opportunity to follow others through viewing comments, or to be followed by others through comments.

### Adding a Video Annotation Module

**FIGURE 4:**
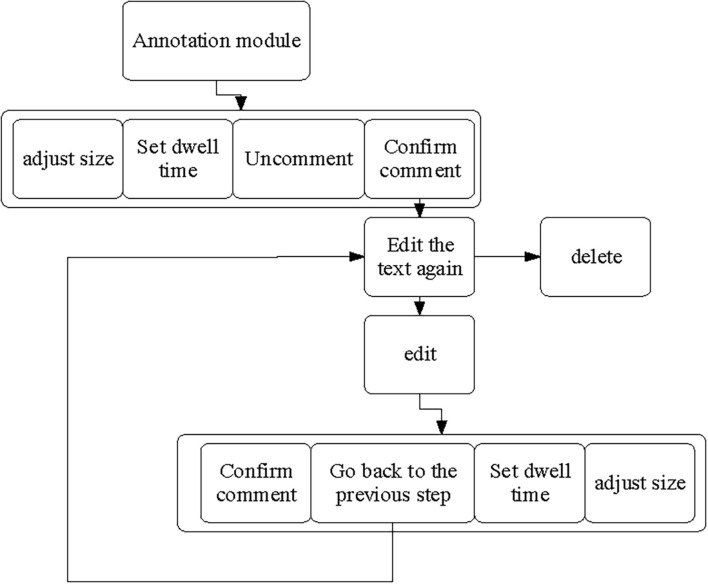
Logical structure of video annotation interactive module.

A video annotation interactive module is added in the participatory art video platform, with Figure 4 presenting the logical structure.

The video annotation interactive module allows users to comment on their ideas and feelings from time nodes on the video screen. Users can express their thoughts and feelings as texts or images when watching videos using the client. When the user clicks the Add Special Effects for Text button on the right side of the video toolbar, the currently playing video will automatically be suspended. Meanwhile, an editable area will be displayed on the video screen for users to input text information and select the size of the annotation and the time to stay in the video. The automatic pause function when adding video reviews provides convenience for users and saves operation time. When the user clicks the Add Image Description button, the user can see the floating photo upload window and set the comment residence time. This information and functionality are the same as text annotations, reducing learning costs and project development costs of users.

### Participatory Video Eye Movement Control Experiment

The eye movement control experiment is conducted to verify the experience and learning effect of online participatory art video platforms and existing video interactive platforms in the market. Eye-tracking is a real-time technology to capture the eye movement of the user, which can provide researchers with physiological information from the eyes of the user in the process of cognition of things, to obtain the test data based on the information. In recent years, the constantly developing eye-tracking technology has become one of the technical means of visual behavior and human behavior in many fields, such as psychology, neuromarketing, neurocognition, user experience, basic research, and market research (Ali et al., 2020; Belanche et al., 2020; Khaleel and Kem, 2020). However, eye-tracking technology is rarely used to evaluate dynamic simulations (Ferreira et al., 2020; Lindsay et al., 2020). Therefore, a participatory art video working scene for dynamic simulations is proposed. The static pages of three working scenes are evaluated by using the portable eye tracker, and the tracker is guided to various situations to obtain the trajectory structure of the heat map of the eye movement and the data at runtime in different situations for comparative study.

The sampling rate of the wearable eye tracker used in this experiment is 100 Hz-Tobii with two glasses. It allows researchers to easily, efficiently, and accurately obtain real eye movement data (Mikelic et al., 2020; Paulina et al., 2020). The experiment is carried out using the Tobii Pro Glasses 2 Controller to observe and mark the changes in the gaze trajectory of the tester. Then Tobii Pro Lab Analyzer is used for data analysis and statistics.

Experimental test sample 1 is the participatory art video platform, and sample 2 is Video Ant, which is a website for academic exchange. Due to the lack of interactive art video products, this experiment selects Video, on which users can upload video directly. Researchers inserted the same video content on both sites before the experiment to minimize the impact of video subjects and comments on the experimental results. The execution time of the work does not include the time for users entering the comment content. Two forms of the participatory video platform used in the experiment have been preconfigured with high-resolution displays of the same size. The subjects are between 22 and 32 years old with no difficulty in operating the computer. Their naked eye or corrected visual acuity is all above 1.2 without other eye diseases.

**FIGURE 5:**
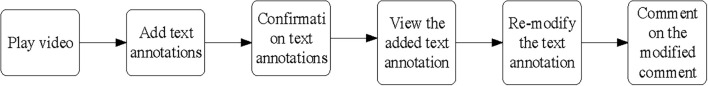
Operation process of participatory video eye movement control experiment.

Figure 5 shows the specific operation process of the experiment after the preparation for the experiment.

After the testee enters the experimental site, the experimental personnel explain the purpose and principle of the test. Under the premise of ensuring the complete understanding of the testee, the experimental personnel guide and help the testee to wear the eye tracker and calibrate. After the calibration, the testee is tested on the participatory art video platform according to the operation process shown in Figure 5. Three groups of different functions are tested, and the experimental data are recorded. After 5 min of rest after the initial experiment, the control experiment is conducted according to the same process, and the test data are collected by experimental personnel.

After the experiment, the testee completes the questionnaire of test experience under the guidance of the personnel to evaluate the user experience (please refer to the Appendix) for details of the questionnaire). The evaluation content includes the degree of love for the two online platforms and the evaluation of the image annotation function. Participants in this experiment are volunteers recruited randomly from the Internet. Due to the constraints of experimental equipment, the test is conducted in different stages at different times in a fixed period. Consequently, the test results may be affected by the period, the quality of equipment, and the profession of volunteers.

## Analysis of the Results of the Control Experiment and Questionnaire Survey

### Analysis of the Results of the Questionnaire Survey on Social Factors

IBM SPSS 25.0 software is used for data analysis, and the obtained reliability analysis results are shown in Table 1.

**TABLE 1:** Questionnaire reliability analysis of social factors.

Project	Correlation coefficient	Item deleted factor α	Cronbach α coefficient
Age level	0.048	0.816	0.812
gender	–0.018	0.827	
Educational background	–0.135	0.819	
Online social networking is more secure than real social networking?	–0.123	0.837	
Do you know anything about artistic composition?	–0.901	0.829	
Do you prefer to use social software for communication?	0.212	0.817	
Can your current social mode meet your social needs?	0.053	0.814	
			

Reliability analysis using IBM SPSS software is commonly used to evaluate the reliability of a questionnaire with Cronbach’s α coefficient as the reference data. Generally, the Cronbach’s α coefficient >0.8 shows a strong reliability of the questionnaire, while the Cronbach’s α coefficient between 0.7 and 0.8 denotes a good reliability. If the Cronbach’s α coefficient is between 0.6 and 0.7, the reliability is general. The Cronbach’s α coefficient <0.6 indicates that the reliability is not enough, and the questionnaire is not reliable. The Cronbach’s α coefficient of this questionnaire is 0.812 (>0.8), showing high reliability.

**FIGURE 6:**
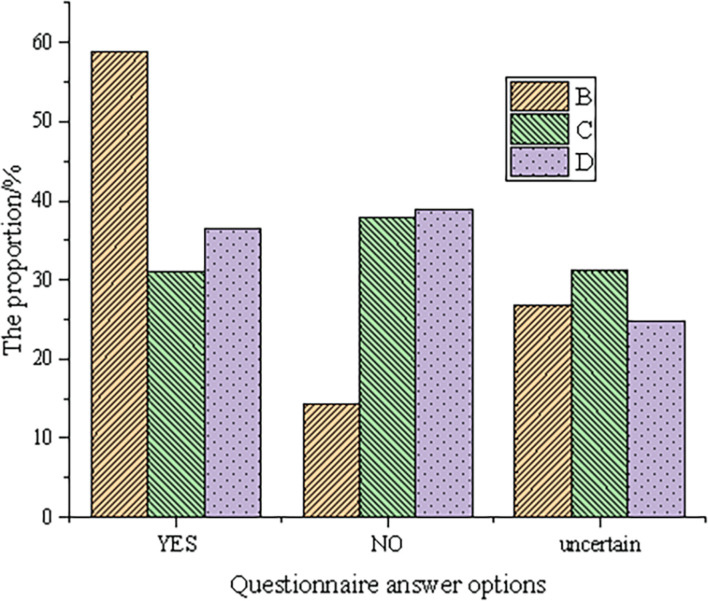
Data of social needs (B, C, and D represent Question B, Question C, and Question D, respectively. Question B: more likely to communicate using social software than face to face. Question C: online socializing is safer than real socializing. Question D: existing ways of social communication can meet social needs).

The data of social needs are shown in Figure 6 based on the answers to the three questions about social situations.

According to the data in Figure 6, approximately 60% of testees are more likely to use social software, while only approximately 13% of testees prefer face-to-face communication. In other words, the number of testees who prefer online platform communication is five times that of offline communication. Evidently, in terms of the demand for communication methods, most people have a great demand for communication with social software, accounting for 58.94%, and 26.74% of research objects are uncertain. In contrast, only a small minority of research objects prefer face-to-face communication, accounting for 14.4%. In Question C, testees who choose the option of YES or NO both occupy 30–40%, but the number of testees who think that offline social is more secure is slightly more. In Question D, there is basically the same proportion of testees choosing YES or NO. In the answer to the above three questions, the number of testees who choose the option of uncertainty is stable at approximately 30%. Therefore, according to the analysis results, it is preliminarily inferred that online artistic social interaction can attract more people to participate in activities than offline social interaction.

### Result Analysis of the Participatory Video Eye Movement Control Experiment

**FIGURE 7:**
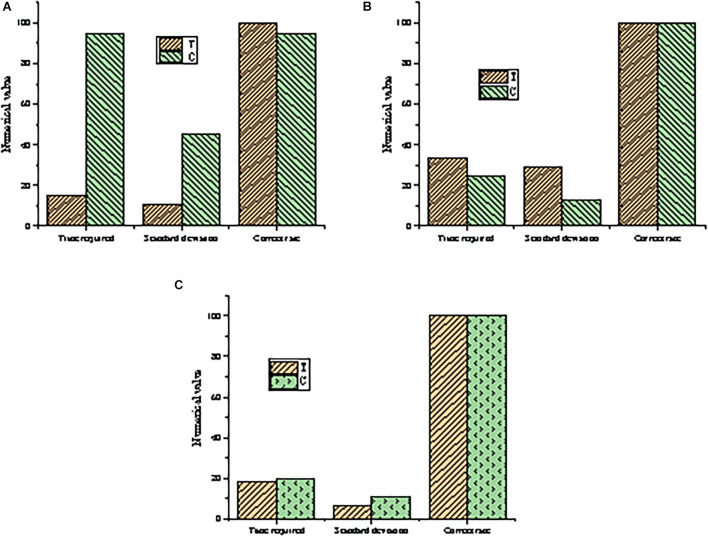
Comparison of testing efficiency (T: test group; C: control group). **(A)** The testing effect of adding video annotation. **(B)** The testing efficiency of modifying annotation. **(C)** The testing effect of commenting on annotation.

The experimental data of the eye movement control experiment are possessed statistically for a comparison between the testing efficiency as shown in Figure 7.

Among Figure 7, Figure 7A shows the testing effect of adding video annotation, Figure 7B shows the testing efficiency of modifying annotation, and Figure 7C shows the testing effect of commenting on annotation. According to the three groups of test results in Figure 7, the operation time of the online participatory art video platform is much shorter than that of the control group. It takes only about 15 s to complete the annotation operation, and the SD is low, indicating that the system performance is stable. The accuracy of the platform also reaches 100%, which is higher than that of the control group. In the second group of tests, the operation time of the online participatory art video platform is basically the same as that of the control group, but the SD is slightly higher. This is caused by the different understanding and modification speed of each test for art video annotation. But the test accuracy of both the control group and the test group reaches 100%. In the third group of tests, the operating time and SD of the online participatory art video platform are slightly better than those of the control group, and the test accuracy reaches 100%. In summary, the online participatory art video platform performs well in the stage of adding video annotations and is basically equal to the control group in the stage of modifying video annotations and commenting on annotations. Overall, its performance is better than that of the control group, especially when adding video annotations, which saves lots of time for the operator and improves the operation efficiency on the premise of ensuring accuracy.

### Result Analysis of the Questionnaire Survey on User Experience

**FIGURE 8:**
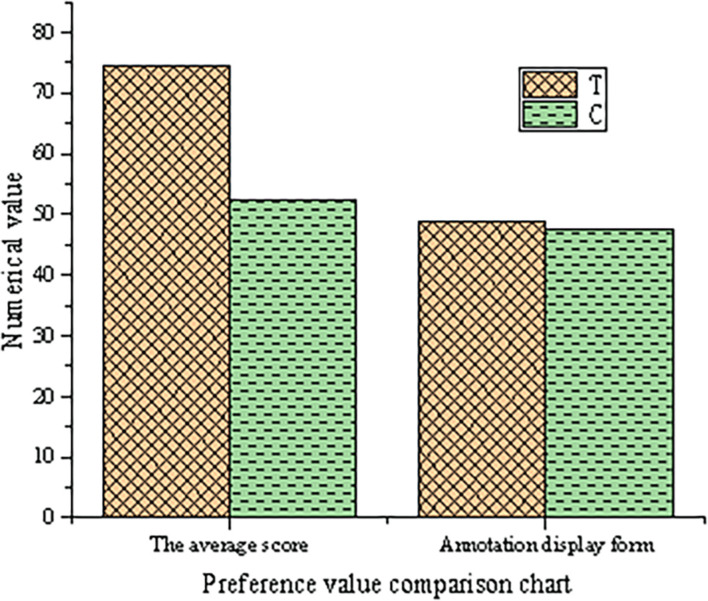
Comparison of the preference value of users.

After the eye movement control experiment, according to the personal test experience of testees, a field investigation is carried out to investigate the customer experience of the online participatory art video platform. According to the scoring of the two art video interaction platforms of testees, a comparison diagram of the preference value of users is drawn as shown in Figure 8.

As shown in Figure 8, in the evaluation of the two art video interaction platforms, the average scoring rate of the online participatory art video platform reaches 75%, compared with 50% in the control group. This indicates that the online participatory art video platform provides a better user experience. From the evaluation of the preference of participants for video annotation styles, the two are basically the same.

**FIGURE 9:**
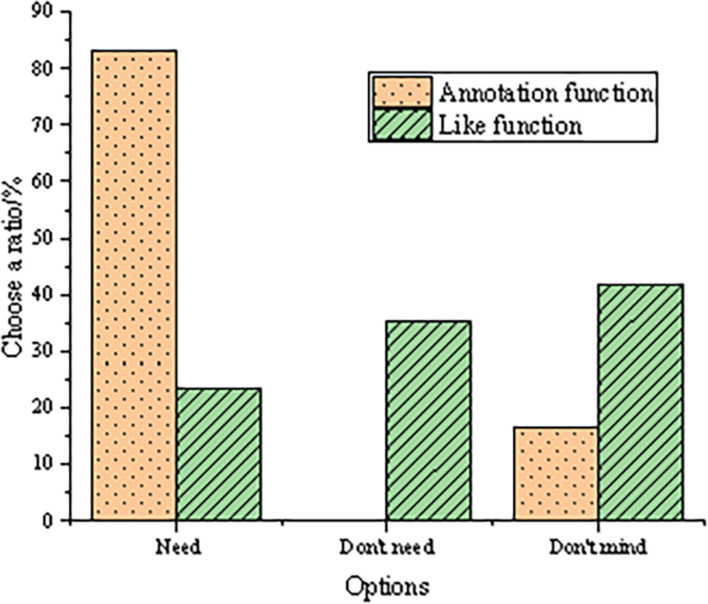
Survey results of the interactive function of video annotation.

According to the evaluation results of participants on the style and function of video annotation, the interactive function of video annotation is analyzed as shown in Figure 9.

From Figure 9, approximately 85% of the testees believe that the image annotation function is needed in the online participatory art video platform, and the rest 15% choose the option of Don’t mind, indicating that the practical image annotation function is expected and loved by the majority in the online participatory art video platform. For the evaluation of the like function, only 23% of the testees think that the online participatory art video platform needs to set the like function, nearly 35% of the testees think that it is not necessary, and the rest more than 40% testees take a neutral attitude. In conclusion, the image annotation function meets the needs of the users on the online participatory art video platform.

In summary, the online participatory art video platform provides a good user experience, meets the needs of users, saves interaction time, and improves interaction efficiency. However, there are still some shortcomings to be improved.

## Conclusion

In this study, the creative combination of an AI algorithm and participatory video is applied to the field of artistic creation and communication. Based on the theory of psychology, a questionnaire is designed to analyze the communication needs of people on art social platforms. According to the results of the survey, the participatory art video HCI platform is constructed, and the preliminary offline test is conducted. Besides, the wearable eye tracker is employed to control experiments of participatory video eye movement from the perspectives of test efficiency and user preference. In this study, the experimental results demonstrate that the participatory art video online client reported provides a satisfactory user experience meeting the user needs, save interaction time, and enhance interaction efficiency. Compared with the past research limited to the traditional human-computer information separation, this study makes a contribution to the application of AI technology in the HCI interface of the participatory art video development platform. The research process can provide new methods and ideas for research in related fields. According to the above limitations of methods and experimental conditions, the results of the experiment may be affected. In this study, due to experimental conditions and financial reasons, the client of the participatory art video HCI platform designed only conducts offline experiments, and the full online operation needs further research in combination with the actual situation, which is not considered in this experiment. In the follow-up work, the HCI interface system will be improved to accelerate the implementation process of the system.

## Data Availability Statement

The raw data supporting the conclusions of this article will be made available by the authors, without undue reservation.

## Ethics Statement

The studies involving human participants were reviewed and approved by the Qilu University of Technology Ethics Committee. The patients/participants provided their written informed consent to participate in this study. Written informed consent was obtained from the individual(s) for the publication of any potentially identifiable images or data included in this article.

## Author Contributions

XL: conceptualization, methodology, and writing—original draft. ML: supervision, data curation, project administration, and resources. Both authors contributed to the article and approved the submitted version.

## Conflict of Interest

The authors declare that the research was conducted in the absence of any commercial or financial relationships that could be construed as a potential conflict of interest.

## Publisher’s Note

All claims expressed in this article are solely those of the authors and do not necessarily represent those of their affiliated organizations, or those of the publisher, the editors and the reviewers. Any product that may be evaluated in this article, or claim that may be made by its manufacturer, is not guaranteed or endorsed by the publisher.

## Appendix

### Social Factors Influencing Factors Questionnaire

1. Personal information
  (1) Your gender A. male B. female
  (2) Your age A. under 35 B. under 50 C. over 51
  (3) Education background A. college degree or below B. bachelor degree C. master degree D. doctor degree
  (4) How many years have you used social software? A. 1–10 years B. 11–20 years C. more than 20 years

2. Related issues
  (1) Do you feel pressure to use social software? A. not at all B. a little stressed C. very stressed
  (2) If social stress is one of your sources of stress, which of the following aspects will make you feel stressed? A. face to face conversation B. the unknown of social software C. no social pressure D. others
  (3) What aspects of social software use do you feel pressure on? A. unknown B. lack of security C. no information guarantee from the other party
  (4) Do you feel that you don’t get along well with friends you know on social software and there are various obstacles? A. often B. sometimes C. never
  (5) Do you often feel bored? A. often B. sometimes C. never
  (6) Do you talk to yourself? A. often B. sometimes C. never
  (7) Have you ever thought about suicide because of pessimism in life? A. often B. sometimes C. never
  (8) Have you ever been unable to sleep because of social problems? A. often B. sometimes C. never
  (9) Are you worried about the stability of your social relationship? A. never worry B. occasionally worry C. often worry
  (10) If possible, do you want to try new ways of socializing? A. I don’t want to. B. I want to
  (11) What do you think of your real social environment? A. very satisfied B. satisfied C. unable to explain clearly D. not very satisfied E. very dissatisfied
  (12) What do you think of your real friend relationship? A. very satisfied B. satisfied C. unable to explain clearly D. not very satisfied E. very dissatisfied
  (13) What do you think of social software regulation? A. very strict B. relatively strict C. general D. relatively loose E. very loose
  (14) What do you think of the use of social software? A. very satisfied B. satisfied C. unable to explain clearly D. not very satisfied E. very dissatisfied
